# Transformative Gamified Binocular Therapy for Unilateral Amblyopia in Young Children: Pilot Prospective Efficacy and Safety Study

**DOI:** 10.2196/63384

**Published:** 2025-01-16

**Authors:** Wenqing Zhu, Shuneng Gu, Jian Li, Jin Lin, Chanling Hu, Rui Liu

**Affiliations:** 1 Department of Ophthalmology, Eye and ENT Hospital of Fudan University, No.83 Fenyang Road, Xuhui District, Shanghai, 200031, China, 86 021-64377134; 2 Key Laboratory of Myopia and Related Eye Diseases, Chinese Academy of Medical Sciences, Fudan University, Shanghai, China; 3 BOKE Digital Health Research Institute, BOKE Medical Technology Co, Ltd, Shanghai, China; 4 State Key Laboratory of Medical Neurobiology and MOE Frontiers Center for Brain Science, Fudan University, Shanghai, China

**Keywords:** amblyopia, binocular treatment, digital therapy, game, stereoacuity, visual acuity

## Abstract

**Background:**

Amblyopia is a common cause of visual impairment in children. Compliance with traditional treatments for amblyopia is challenging due to negative psychosocial impacts. Recent shifts in amblyopia treatment have moved from suppressing the dominant eye to enhancing binocular visual function. Binocular digital therapy has become a promising approach.

**Objective:**

The aim of this study was to evaluate the effects of binocular gamified digital therapy on visual acuity and stereoacuity (SA) in children with unilateral amblyopia.

**Methods:**

This pilot prospective study enrolled 11 children aged 4-6 years with unilateral amblyopia. Following at least 8 weeks of refractive correction, participants underwent binocular gamified digital therapy for 60 minutes per day, 5 days a week. The therapy used a roguelike shooting game delivered under binocular conditions through two independent channels with a real-time artificial intelligence visual engine. Assessments of distance visual acuity (DVA), near visual acuity (NVA), and SA were conducted at baseline and again at 4, 8, and 12 weeks.

**Results:**

At 12 weeks, the following significant improvements were noted: amblyopic eye DVA improved by 1.0 line (*P*=.01; *d*=0.77), binocular DVA improved by 0.7 lines (*P*=.006; *d*=1.00), and SA improved by 0.3 logarithm (log) arcseconds (*P*=.01; *d*=0.97). At 8 weeks, improvements included amblyopic eye DVA by 0.9 lines (*P*=.046; *d*=1.00) and SA by 0.28 log arcseconds (*P*=.02; *d*=0.90). No significant adverse events were observed, although one participant developed progressive esotropia.

**Conclusions:**

Binocular gamified digital therapy is effective and safe for improving visual outcomes in children aged 4-6 years with unilateral amblyopia.

## Introduction

Amblyopia refers to a unilateral or bilateral decrease of vision caused by abnormal vision development in childhood. It is a common cause of visual impairment in children, significantly affecting their social environment and quality of life. Studies indicate that amblyopia affects 0.7%-2.6% of children aged 30‐71 months and 1%-5.5% of older children [1-3]. Anisometropia is linked to a higher risk of developing amblyopia, with about one-third of children with amblyopia displaying this condition [4]. Additionally, 19%-50% of cases of unilateral amblyopia are associated with strabismus, which increases the risk of amblyopia by 2.7-18 fold [5]. Traditional treatments include refractive correction [6,7] and clinical therapies like part-time patching or atropine penalization of the dominant eye [8,9]. However, compliance with these treatments is challenging, with only about 50% of children adhering to patching regimens due to negative psychosocial impacts [10]. Patching also fails to enhance binocular vision effectively and may disrupt motion processing [11], reading fluency [12], fixation [13], and visual perception [14].

Recent shifts in amblyopia treatment have moved from suppressing the dominant eye with monocular interventions to enhancing binocular visual function. Binocular therapy has thus become a promising approach [15,16]. Many children with amblyopia have a functional binocular visual system and cerebral cortex that can facilitate vision in the amblyopic eye by reducing the contrast sensitivity in the dominant eye. This concept underpins the clinical use of binocular vision therapy [17,18]. Gamification has been reported to support adherence to therapeutic amblyopia treatment and improve visual-perceptual defects in children [19,20]. The Vision Planet Visual Training System in this study, a gamified home-based medical software, treats amblyopia under binocular conditions by presenting separate visual stimuli to each eye. The software features an in-game joystick and a shoot button that allow the user to control a character, aiming to improve gaze and binocular fusion through interactive gameplay.

In this study, we present an initial evaluation of a novel binocular digital therapy that employs active viewing with feedback, designed to strengthen the amblyopic eye without the participants’ awareness. The objective of this prospective pilot study was to assess the efficacy and safety of this therapy in children with unilateral amblyopia.

## Methods

### Participants

The study was a pilot, prospective, single-arm proof-of-concept trial conducted at the Eye and ENT Hospital of Fudan University in Shanghai, China, from June 2023 to January 2024. A total of 11 participants were enrolled.

The primary inclusion criteria were as follows: (1) children aged between 4-7 years; (2) diagnosed with anisometropic, strabismic, or combined mechanism amblyopia; (3) visual acuity (VA) in the amblyopic eye ranging from 20/32 (0.2 base 10 logarithm of the minimum angle of resolution [logMAR]) to 20/200 (1.0 logMAR); (4) best corrected visual acuity (BCVA) in the dominant eye of 20/40 (0.3 logMAR) or better for children aged 4-5 years, and 20/32 (0.2 logMAR) or better for children 5 years or older; (5) an interocular difference of ≥2 logMAR lines (≥10 letters); (6) consistent spectacle wear for at least 8 weeks, if required; (7) a pause in patching or any binocular therapy for at least 2 weeks; (8) partially accommodative esotropia with ≤10 prism diopters (PDs) of near deviation postrefractive correction or well-controlled intermittent exotropia. Major exclusion criteria included (1) a spherical equivalent of ≤–0.5 diopter in either eye, (2) any other condition potentially causing reduced BCVA, (3) severe developmental delays affecting participation in treatment or evaluation, (4) noncompliance with spectacle wear, (5) a history of light-induced seizures, (6) use of rigid gas permeable contact lenses, (7) any reported anatomical ocular anomalies, and (8) prior intraocular or strabismus surgery.

### Ethical Considerations

This study involving human subjects received ethical approval from the institutional review board (IRB) at the Eye and ENT Hospital of Fudan University under approval number 2023061. The research adhered to all relevant ethical guidelines for studies involving humans, medical records, patient information, and secondary data analyses. No exemptions or waivers were applied for this study, and the ethics approval was granted in accordance with institutional requirements. Informed consent was obtained from all participants prior to their inclusion in the study. Participants were informed about the purpose, procedures, potential risks, and their right to withdraw at any point without consequence. For secondary analyses of existing data, the original informed consent or IRB approval covered the use of this data for further analysis without requiring additional consent. All data collected in this study was anonymized to ensure the privacy and confidentiality of the participants. Personal identifiers were removed, and data was stored securely in compliance with institutional privacy protocols. In cases where data could potentially be linked to individuals, appropriate measures, including encryption and access control, were in place to protect participant information. Participants in this study received compensation for their time and involvement. The amount and type of compensation were clearly communicated to all participants prior to their participation that they will receive a transportation allowance of approximately US $80 in total (about $20 per visit). Details of compensation were outlined in the informed consent form, ensuring transparency and fairness in the compensation process. No identifiable features of research participants are visible in any images included in the manuscript or supplementary materials.

### Gamified Binocular Therapy

The Vision Planet Visual Training System is a gamified medical software developed using Unity version 2020.3.48 (Unity Technologies) and features a roguelike shooting game. Roguelike games are characterized by minimalistic graphics and random generation of contents, such as items, enemies, and level layouts. The system employed the classic mechanics, dynamics, and aesthetics model as a framework, which involves operating characters to shoot and defeat monsters. The dynamic model involved players combining different equipment and buffs to construct personalized play styles to battle with monsters in various planets, which enabled players to experience the aesthetics of discovery when uncovering unknown elements, challenges when facing powerful bosses, and immersion in the storyline through fantasy. The system used the Berlin Interpretation to instruct the roguelike mechanism, including factors like tactical challenge, random environment generation, permadeath, and resource management. It employed a novel approach to treat amblyopia by delivering visual stimuli through two independent channels, presenting them separately to each eye. Vision Planet was a newly developed serious game and certified as a Class II medical device by the National Medical Products Administration in mainland China (identifier: zhexiezhuzhun20212210379).

**Figure 1.**
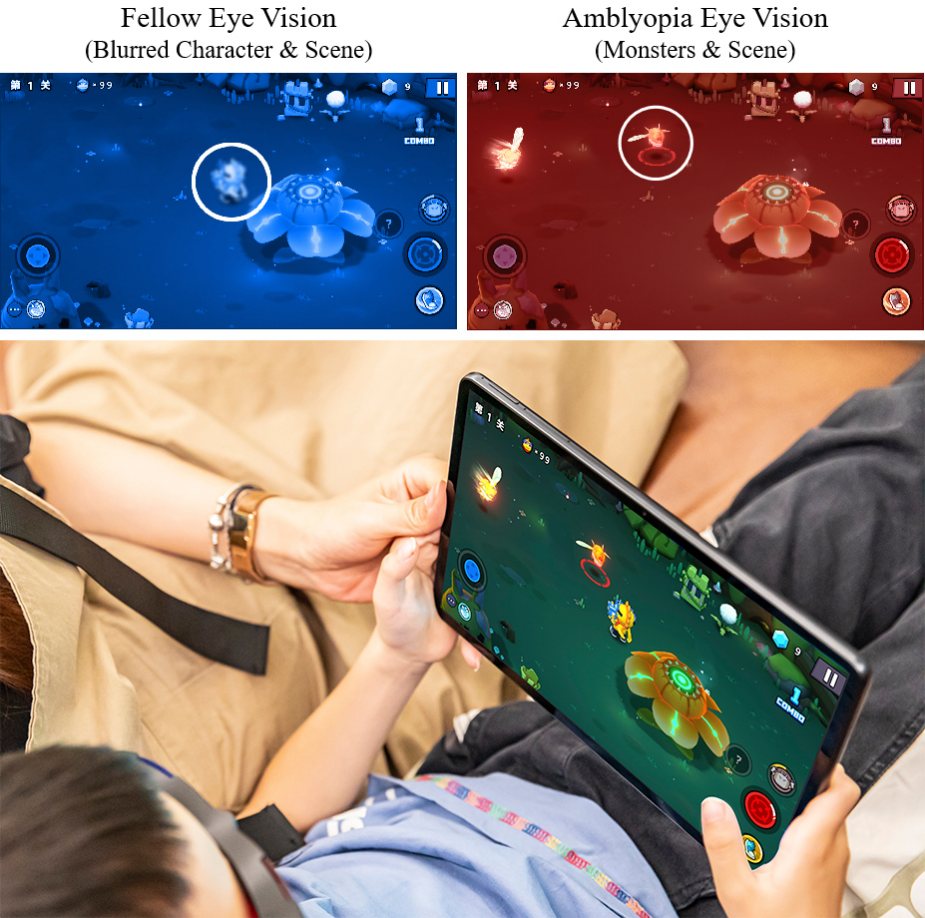
Binocular treatment setup. The top illustration shows a red-blue dichoptic game frame with a blurred character visual intended for the fellow eye and a monster visual for the amblyopic eye. The bottom image is a participant during a treatment session.

The treatment setup included (1) an 11-inch Huwawei MatePad (Model DBR-W00) that operated the software and presented the visual stimuli, (2) anaglyph glasses that separated the visual stimuli and applied a monocular Gaussian blur to the fellow eye channel, and (3) the Vision Planet gamified medical software. The software automatically adjusted the magnitude of the blur based on the baseline VA difference between the two eyes, with a greater disparity resulting in a higher blur intensity. During the sessions, character visuals were directed to the fellow eye and monster visuals to the amblyopic eye. Participants controlled the character’s movements and attacks on the monsters using a virtual joystick and shoot button, aiming to achieve simultaneous gaze and binocular fusion, as illustrated in Figure 1.

**Figure 2.**
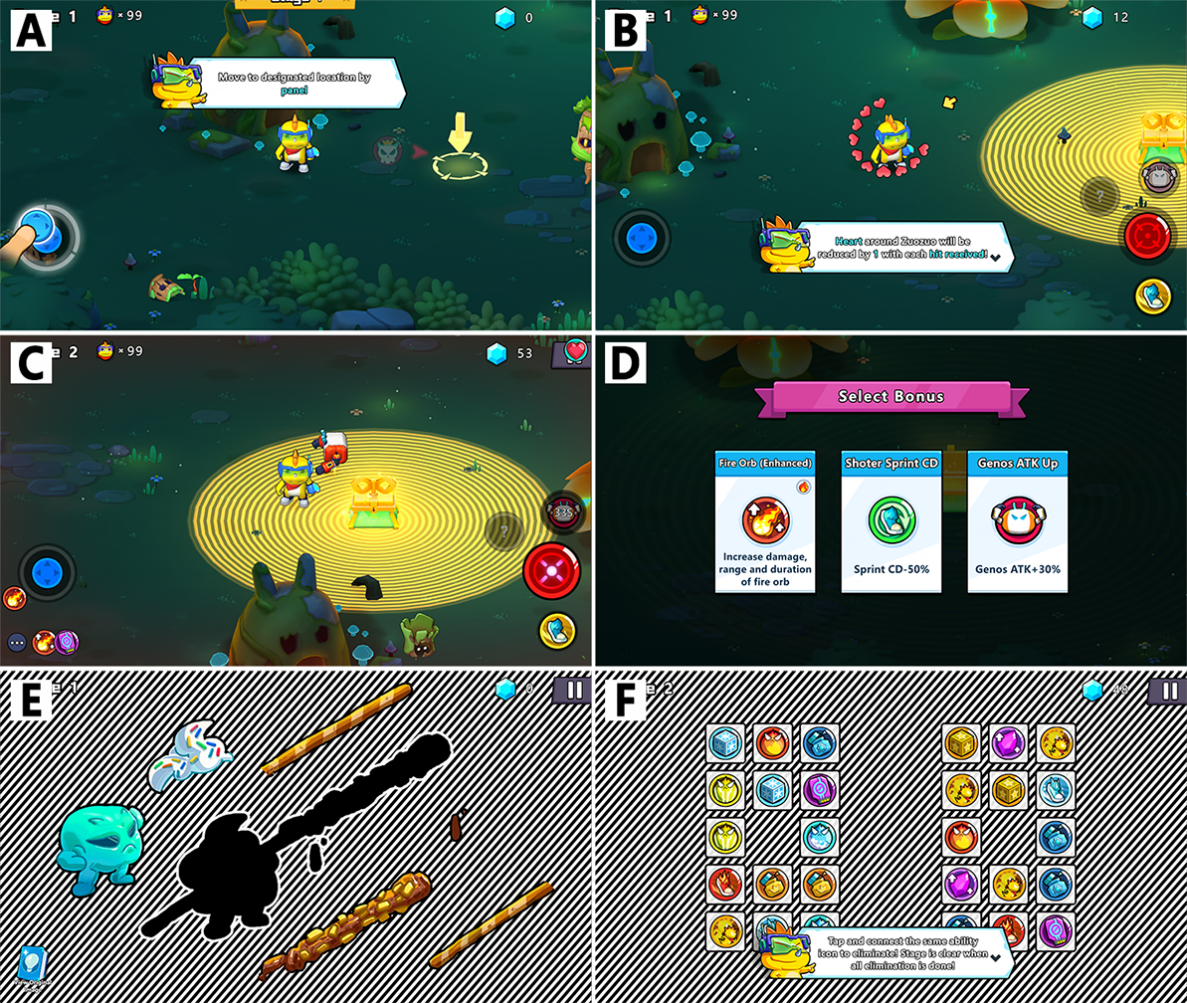
Training journey. The training journey in Vision Planet includes a tutorial level, main game levels, and bonus levels. (A and B) A structured tutorial level was designed to acquaint users with the basic operational procedures at the first training session. (C and D) In main game levels, users were rewarded with a treasure box upon successfully clearing a level, which contained a selection of 3 buffs. (E and F) Users progressed to a bonus level after completing 3 regular levels. The bonus gameplay was distinct and less challenging to offer a rhythmic break from the main game’s intensity.

During the first Vision Planet training session, users were guided through a tutorial level that familiarized them with basic operational procedures, including movement, attack mechanics, injury responses, level completion strategies, and enemy attack patterns. In the primary game levels of Vision Planet, users should have eliminated all monsters to complete the level. Upon successful clearance, a treasure box was awarded which contained 3 buffs. The interfaces of the tutorial level, main game levels, and bonus levels are illustrated in Figure 2.

**Figure 3.**
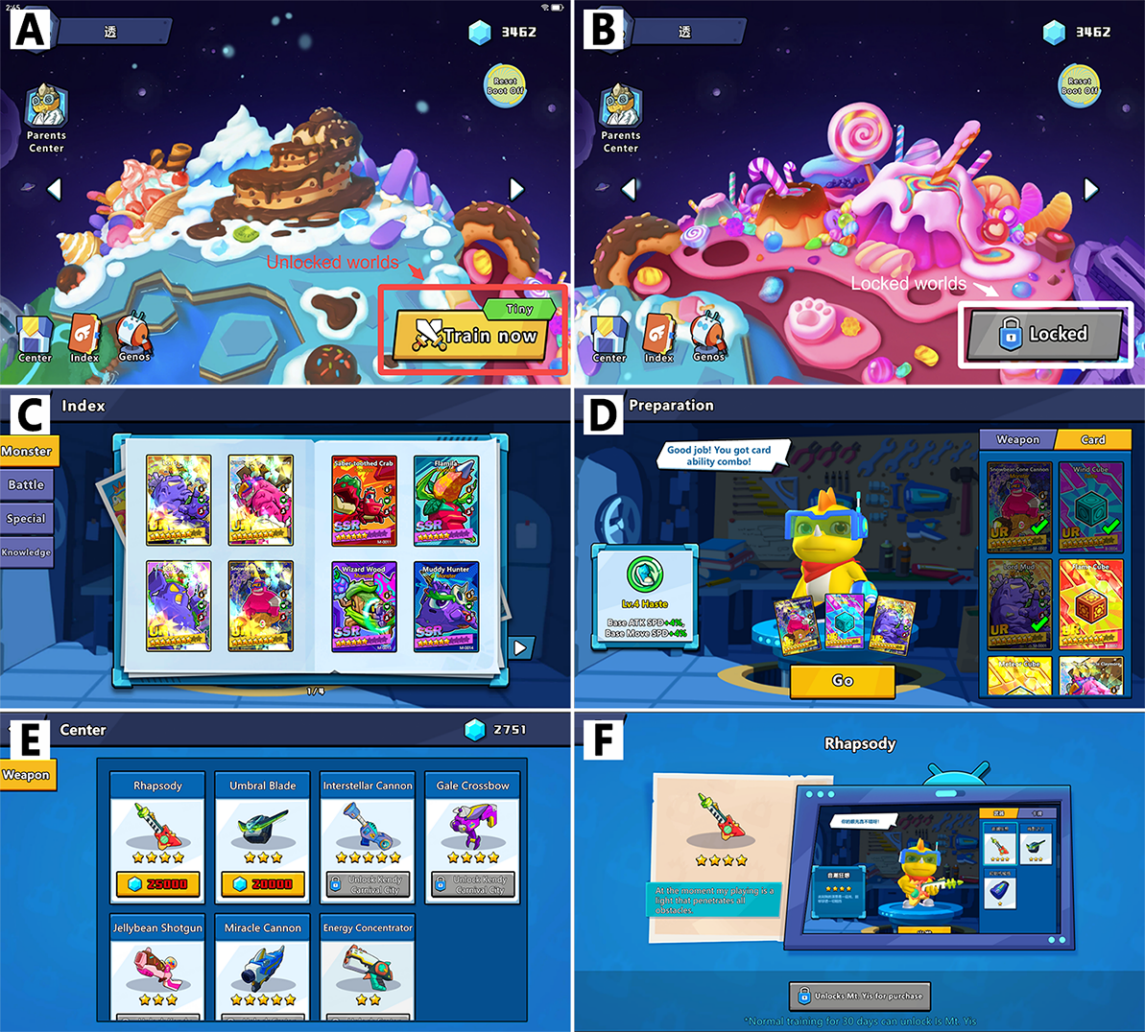
Incentive system. The incentive system mainly consists of unlockable levels, a gacha system, and an in-game store. (A and B) Vision Planet comprised 5 worlds with only the first available from the start, and others unlocked after meeting daily training time requirements for set days. (C and D) Users earned energy stones by defeating bosses for gacha draws, getting cards that boosted battles. (E and F) Users defeated monsters to earn gems for purchasing unique weapons, which could be equipped in the preparation interface to customize their training experience.

Vision Planet comprised 5 worlds that could be unlocked by meeting daily training goals, offering new elements and motivating users to continue their training. Boss level completion was rewarded with energy stones for card draws in the gacha system. Acquired cards could be strategically deployed for buffs in battles. Gems earned from defeating monsters could be used to purchase unique weapons from the store. Users had the option to select and equip their preferred weapon in the preparation interface (Figure 3).

**Figure 4.**
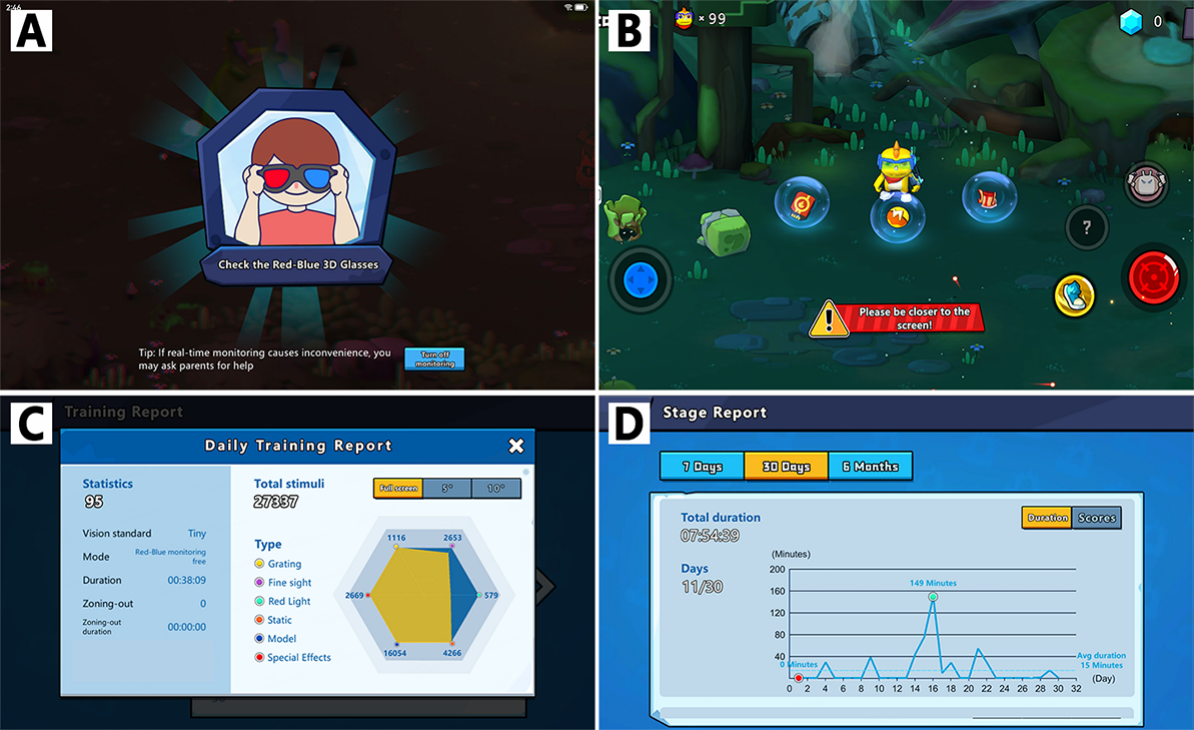
Back-end monitoring included supervised training and a summary report. (A and B) The visual engine monitored and prompted users to maintain a 40 cm distance and wear anaglyph glasses correctly for effective training. (C and D) A parent center and developer back end offered daily training metrics to help parents and providers monitor progress and prevent gaming addictions.

Using a real-time artificial intelligence visual engine, Vision Planet was capable of continuously monitoring and reminding participants to keep their eyes parallel to the screen at a distance of about 40 cm, while ensuring that participants wore the anaglyph glasses properly throughout the training session to guarantee the standard conduction of the intervention. Images were captured in real time by the built-in camera of the training tablet, and processed through the visual engine (algorithm) of the Vision Planet software. Vision Planet also calculated daily training metrics, including the duration of play and the scores, which could be shown in reports for various training cycles (Figure 4).

### Study Schedule

After a minimum of 8 weeks of refractive correction, participants underwent binocular digital therapy for 60 minutes daily, 5 days a week. The primary outcomes measured were DVA, NVA, and SA. DVA was assessed using an electronic VA system with current refractive correction as necessary, following the Pediatric Eye Disease Investigator Group Amblyopia Treatment Study protocols, which use Lea symbols with crowded optotypes. NVA was tested at a distance of 40 cm using the Amblyopia Treatment Study’s near acuity test, which included flip cards featuring single-surrounded Lea symbol charts [21]. SA was evaluated using the Titmus SA chart (by Stereo Optical) with current spectacle corrections as required. Ocular alignment was determined through the simultaneous prism and cover test. All assessments were performed by the same study-certified examiner and were scheduled at 4, 8, and 12 weeks. Patient compliance and satisfaction with the binocular digital therapy were evaluated at the week 8 visit using the Vision Planet Questionnaire (Multimedia Appendix 1).

### Statistical Analysis

Data was analyzed using the SPSS version 25.0 software (IBM Corp). Continuous variables were presented as mean and SD, and categorical variables were reported as frequency and percentage. Stereopsis measurements were transformed to a natural logarithmic scale for statistical analyses. Differences in amblyopic eye and binocular DVA, NVA, and SA between the baseline and follow-up visits at 4, 8, and 12 weeks were evaluated using paired 2-tailed *t* tests. The effect size was calculated using a Cohen *d*, determined by dividing the mean difference by the SD of the differences. *P* values <.05 were considered statistically significant.

## Results

### Participant Characteristics

The study involved 11 children with amblyopia aged 4‐6 (mean 5.6, SD 0.7) years. The cohort included 9 participants with anisometropia and 2 with combined mechanism amblyopia. All participants had received refractive correction at least 8 weeks prior to the study. In total, 8 participants had previously undergone patching and paused at least 2 weeks before the study. No participants had prior exposure to any other binocular therapies. Baseline characteristics of the participants are detailed in Table 1.

**Table 1.** Baseline characteristics of the participants.

Participant number	Age (years)	Sex	Amblyopic eye	Amblyopic eye VA^a^ (logMAR^b^)	Fellow eye VA (logMAR)	Spherical refraction (amblyopic eye)	Spherical refraction (fellow eye)	Type of amblyopia	Tropia	Refractive correction duration (years)	Previous patching treatment	Initial stereoacuity (arcseconds)
1	4.8	F^c^	OS	0.3	0.1	+5.25	+2.50	C^d^	10 E(T)^e^	2.5	Yes	800
2	5.9	F	OS	0.4	0	+2.75	+1.25	A^f^	—^g^	1.5	Yes	400
3	6.1	M^h^	OD	0.3	0	+8.00	+1.50	A	—	2.5	Yes	400
4	4.4	F	OS	0.2	0	+4.75	+1.25	C	30 X(T)^i^	1.5	Yes	800
5	6.9	M	OD	0.6	–0.1	+2.75	+1.00	A	—	1	Yes	400
6	4.9	F	OD	0.3	0	+6.50	+2.75	A	—	1.5	No	200
7	5.8	F	OS	0.4	0.1	+5.50	+4.00	A	—	0.2	No	60
8	6.3	M	OS	0.4	–0.1	+5.75	+2.00	A	—	0.2	No	60
9	5.9	M	OD	0.2	0	+6.00	+4.00	A	—	1.5	Yes	80
10	5.7	M	OS	0.4	0	+7.25	+3.25	A	—	1	Yes	400
11	5.3	M	OS	0.2	0	+4.50	+3.25	A	—	0.5	Yes	50

a VA: visual acuity.

b logMAR: base 10 logarithm of the minimum angle of resolution.

c F: female.

d C: combined.

e E(T): intermittent esotropia.

f A: anisometropic.

g Em dash indicates that participant did not have tropia.

h M: male.

i X(T): intermittent exotropia.

### Visual Acuity and Stereoacuity

Before the intervention, the average BCVA of the amblyopic eye was 0.34 (SD 0.12) logMAR for DVA and 0.33 (SD 0.17) logMAR for NVA. The average binocular BCVA was 0 (SD 0.06) logMAR for DVA and 0.02 (SD 0.08) logMAR for NVA. The average SA was 2.34 (SD 0.46) log arcseconds. The DVA for the amblyopic eye improved to 0.31 (SD 0.13) logMAR at 4 weeks, 0.25 (SD 0.10) logMAR at 8 weeks, and 0.24 (SD 0.09) logMAR at 12 weeks. Statistically significant improvements were noted in the DVA of the amblyopic eye from baseline, with an increase of 0.9 lines at 8 weeks (*P*=.046; *d*=1.00) and 1.0 line at 12 weeks (*P*=.01; *d*=0.77), while no significant change was observed at 4 weeks (*P*=.34). Binocular BCVA for DVA improved to –0.1 (SD 0.08) logMAR at 4 weeks, 0 (SD 0.07) logMAR at 8 weeks, and −0.07 (SD 0.07) logMAR at 12 weeks, corresponding to a significant enhancement of 0.7 lines at 12 weeks (*P*=.006; *d*=1.00), with no significant changes at 4 weeks (*P*=.10) or 8 weeks (*P*=.19).

The SA improved to 2.16 (SD 0.46) log arcseconds at 4 weeks, 2.06 (SD 0.41) log arcseconds at 8 weeks, and 2.04 (SD 0.39) log arcseconds at 12 weeks. Statistically significant improvements were noted in SA from baseline, with improvements of 0.28 log arcseconds at 8 weeks (*P*=.02; *d*=0.90) and 0.3 log arcseconds at 12 weeks (*P*=.01; *d*=0.97), while no significant change was observed at 4 weeks (*P*=.06). For the NVA, no statistically significant changes were observed for either the amblyopic eye or binocularly at any time point. The NVA for the amblyopic eye improved to 0.32 (SD 0.13) logMAR at 4 weeks (*P*=.68), 0.28 (SD 0.13) logMAR at 8 weeks (*P*=.27), and 0.24 (SD 0.09) logMAR at 12 weeks (*P*=.06). Binocular BCVA for NVA was recorded as 0.05 (SD 0.07) logMAR at 4 weeks (*P*=.27), 0 (SD 0.05) logMAR at 8 weeks (*P*=.34), and 0 (SD 0.06) logMAR at 12 weeks (*P*=.17).

**Figure 5.**
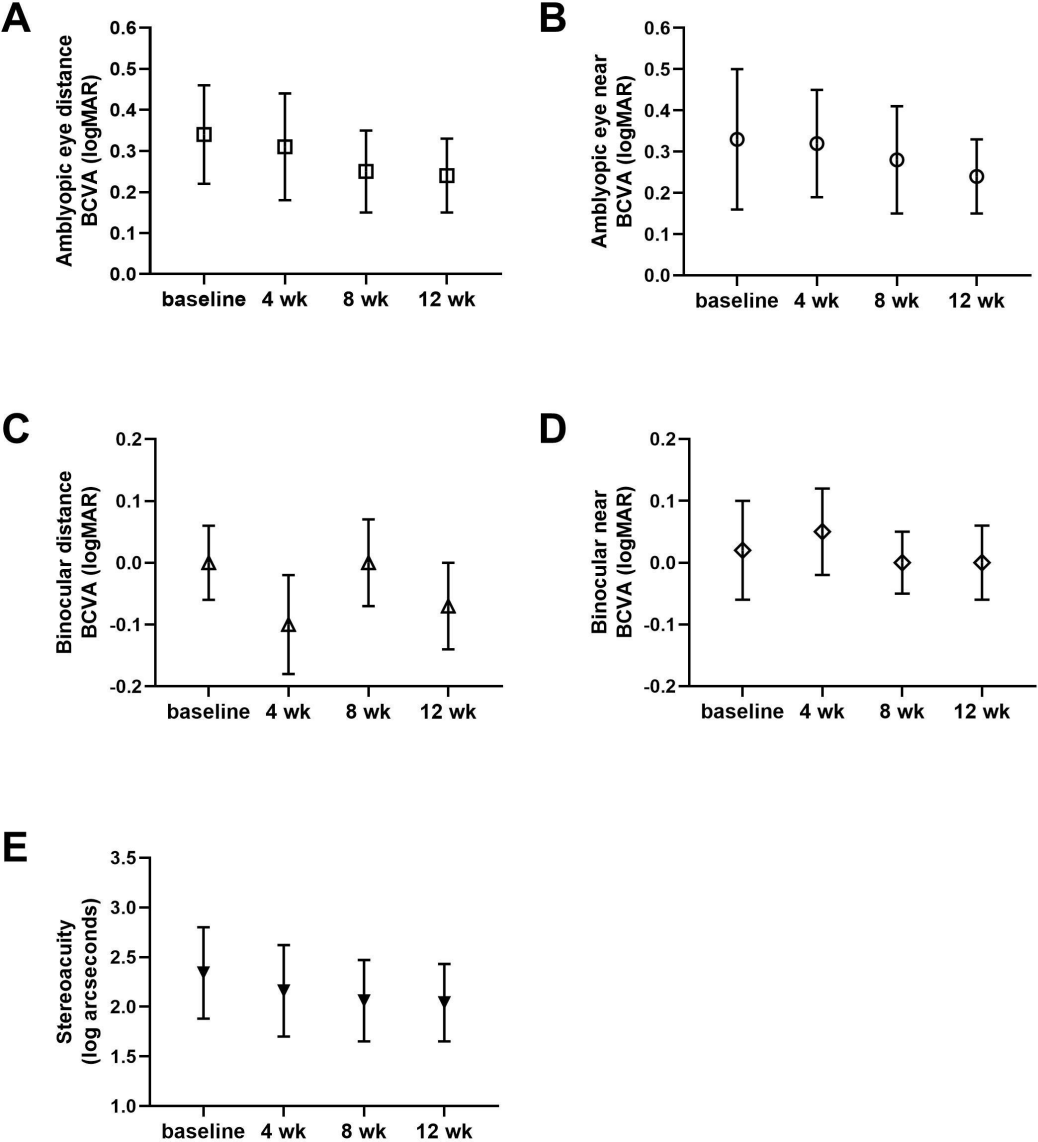
Improvement in visual acuity and stereoacuity. Best corrected visual acuity (BCVA) and stereoacuity were examined at 4 study time points—baseline, 4 weeks, 8 weeks, and 12 weeks. These are presented for (A) distance amblyopic eye BCVA, (B) near amblyopic eye BCVA, (C) distance binocular BCVA, (D) near binocular BCVA, and (E) stereoacuity. Error bars represent SD. log: logarithm; logMAR: base 10 logarithm of the minimum angle of resolution.

**Figure 6.**
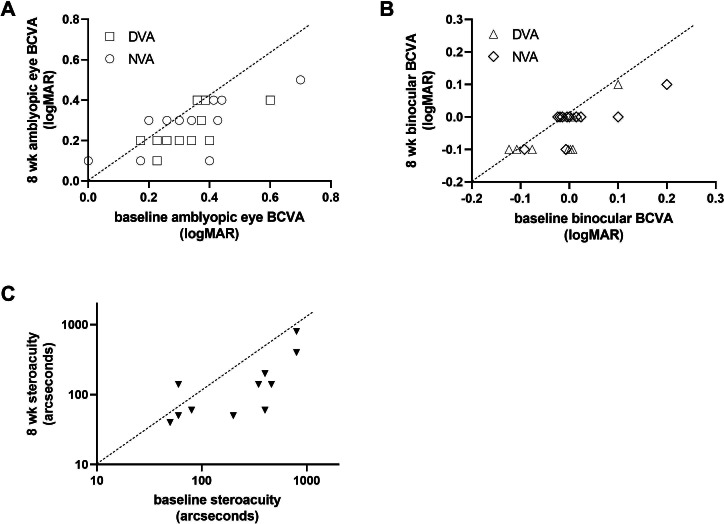
Improvement in BCVA and stereoacuity at the week 8 follow-up visit, as a function of baseline measurements. Individual data is depicted for the measurements of (A) distance and near amblyopic eye BCVA, (B) distance and near binocular BCVA, and (C) stereoacuity. Data under the diagonal unity line indicated amelioration. A value of 800 arcseconds was arbitrarily assigned if subjects failed the Titmus test. BCVA: best corrected visual acuity; DVA: distance visual acuity; NVA: near visual acuity.

Statistical results for VA and SA across all time points are shown in Table 2. Improvements in VA and SA at all time points are depicted in Figure 5. The progress of each participant in BCVA for the amblyopic eye, binocular BCVA, and SA at the week 8 follow-up is illustrated in Figure 6.

**Table 2.** Pairwise comparisons and Cohen *d*.

	Mean difference	Standardized mean difference(95% CI)		Cohen *d*	*t* value	*P* value
Distance visual acuity of the amblyopic eye						
Baseline vs 4 wks	0.02	0.06 (–0.02 to 0.06)		0.50	1.00	.34
Baseline vs 8 wks	0.06	0.09 (0-0.13)		1.00	2.28	.046
Baseline vs 12 wks	0.12	0.13 (0.03-0.21)		0.77	2.95	.01
Near visual acuity of the amblyopic eye						
Baseline vs 4 wks	0.01	0.07 (–0.04 to 0.06)		0.14	0.43	.68
Baseline vs 8 wks	0.05	0.13 (–0.04 to 0.13)		0.38	1.17	.27
Baseline vs 12 wks	0.08	0.13 (0-0.17)		0.69	2.17	.06
Binocular distance visual acuity						
Baseline vs 4 wks	0.04	0.07 (–0.01 to 0.08)		0.43	1.79	.10
Baseline vs 8 wks	0.03	0.06 (–0.02 to 0.07)		0.50	1.40	.19
Baseline vs 12 wks	0.05	0.05 (0.02-0.09)		1.00	3.46	.006
Binocular near visual acuity						
Baseline vs 4 wks	–0.04	0.10 (–0.11 to 0.03)		0.30	–1.17	.27
Baseline vs 8 wks	0.02	0.06 (–0.02 to 0.06)		0.50	1.00	.34
Baseline vs 12 wks	0.02	0.04 (–0.01 to 0.05)		0.50	1.49	.17
Stereoacuity						
Baseline vs 4 wks	0.17	0.26 (–0.01 to 0.35)		0.69	2.16	.06
Baseline vs 8 wks	0.27	0.31 (0.06-0.48)		0.90	2.89	.02
Baseline vs 12 wks	0.30	0.31 (0.09-0.51)		0.97	3.18	.01

### Satisfaction Questionnaires and Compliance

The exit questionnaire revealed that 91% (10/11) of participants found Vision Planet easy or very easy to use and 64% (7/11) would prefer Vision Planet over patching for future amblyopia treatments. Detailed satisfaction data are available in Multimedia Appendix 1. The overall training completion rate for all participants at the week 8 follow-up was 98.5% (range: 93-114.5%), with 82% (9/11) of participants completing more than 90% of the prescribed training time. Detailed compliance data are shown in Multimedia Appendix 2.

### Adverse Events

Participants were consistently monitored for adverse events such as diplopia, new or worsening eye heterotropia, deteriorative VA, or unexpected adverse events. No significant adverse events were observed, although one participant developed progressive esotropia. This participant had a near and far deviation of 10 PD esotropia with glasses at baseline, which increased to 20 PD esotropia with glasses by 12 weeks. The amblyopic eye DVA remained stable at 0.3 logMAR from baseline to 12 weeks. Stereoacuity improved from 800 arcseconds at baseline to 400 arcseconds at 12 weeks. Deviations remained stable at 4 and 8 weeks, but progressive esotropia was noted at the last follow-up. A recycloplegic refraction was immediately performed and full hyperopia spectacles were prescribed.

## Discussion

### Principal Findings

This pilot study confirmed the efficacy and safety of binocular digital therapy for treating anisometropic amblyopia in children aged 4-6 years. We observed improvements in amblyopic eye DVA and SA following this treatment. Apart from one case of progressive esotropia, no significant adverse events were reported.

Previous research on binocular therapy has documented VA enhancements through the use of dichoptic video games, contrast-adjusted films, and shutter glasses during video or interactive tasks [22,23]. Our findings add to the existing literature by demonstrating significant improvements in both DVA of the amblyopic eye and binocular DVA using the Vision Planet binocular treatment. However, another study involving children aged 7‐12 years did not show any improvement in amblyopic eye VA at 16 weeks [24], possibly due to the older age of the participants. In contrast, a multicenter randomized controlled trial reported a 1.8-line improvement in amblyopic eye VA with digital treatment compared to a 0.8-line improvement with continued spectacle use alone at 12 weeks, in children aged 4‐7 years [25]. Recently, Wygnanski-Jaffe et al [26] found a 2.8-line improvement in BCVA of the amblyopic eye in children aged 4‐9 years using a binocular eye tracking–based home treatment (CureSight) over 16 weeks, showing noninferior results compared to patching treatment, which achieved a 2.3-line improvement. Compared to the 1.0-line improvement in our study, the significant results in their study could be attributed to the longer duration of training (90 min/d for 16 weeks). Additionally, our prior research analyzed the dosing schedule of CureSight binocular treatment, revealing that more frequent treatment (5 days per week) was more effective than less frequent sessions (3 days per week), with improvements of 1.8 lines versus 1.5 lines for DVA and 2.9 lines versus 1.7 lines for NVA, respectively [27]. This suggests that a longer duration for binocular treatment may be more effective in treating amblyopia and the treatment in our study (60 min/d, 5 d/wk, for 12 weeks) may not have been sufficient to achieve optimal results.

Numerous studies have supported the improvement in SA with binocular treatment for amblyopia [18,28,29], while others have reported no significant changes [24,25,30]. In this study, we observed statistically significant enhancements in SA from baseline by 0.28 log arcseconds at 8 weeks and by 0.3 log arcseconds at 12 weeks with Vision Planet treatment. Similarly, our prior study with CureSight treatment showed significant improvement in SA by 0.38 log arcseconds at 12 weeks. Research indicates that binocular therapy could substantially benefit the restoration of binocular function. SA is closely linked with the depth of suppression but does not correlate with decreases in VA [31]. By addressing suppression and enhancing contrast balance, binocular digital therapy has shown potential as an effective alternative for treating amblyopia [30]. The success of binocular digital treatments may be closely related to the age of the participants and the engaging nature of the treatment content. Young children may benefit more from SA improvement due to cortical plasticity. Importantly, the content of binocular treatments should be engaging enough to maintain children’s attention throughout the training period.

### Comparison to Other Gamification Therapies

The falling blocks game is a commonly used gamification therapy for amblyopia. Participants score points in the game by moving the falling blocks to form complete lines of blocks. Holmes et al [32] reported that the mean amblyopic eye VA improved by 1.05 lines in the binocular falling blocks game group and 1.35 lines in the patching group at 16 weeks among children aged 5 to <13 years, which was similar to the DVA improvement observed at 12 weeks in our study (1.0 line). Gao et al [33] used a home-based binocular falling blocks game to treat amblyopia, reporting that the mean amblyopic eye VA improved by 0.6 lines from baseline in the binocular game group and 0.7 lines in the placebo game group at 6 weeks, with no statistical improvement noted. The limited effect of the gamified binocular therapy may be attributed to the short duration of the treatment.

Another gamification therapy for amblyopia is the Dig Rush game, an action-oriented adventure game with 42 levels in which miners dig for gold. Kelly et al [30] reported a mean improvement of 1.5 lines with 2 weeks of binocular Dig Rush game treatment in children aged 4-10 years, which was greater than the improvement observed in those treated with part-time patching (0.7 lines). However, the Pediatric Eye Disease Investigator Group found no benefit to VA or SA in amblyopia from binocular Dig Rush game treatment for children aged 7-12 years at 8 weeks, which might be attributed to the older age of the participants [34].

Finally, Gambacorta and colleagues [35] used the mirror stereoscope to achieve alignment and dichoptic games to treat amblyopia. The children were instructed to move around, collect health points, and tag robot opponents by aligning the fixation scope onto the center of the robot and clicking the mouse to activate the pointer tool. Following 20 hours of training, the VA of the amblyopic eye improved by 1.4 lines for the dichoptic group and by 0.6 lines for the monocular group. However, this therapy needed to be conducted in the lab, which increased the time and cost.

Compared to the falling blocks game and the Dig Rush game, the Vision Planet system incorporates a roguelike shooting game element, adding an entertaining dimension to the therapeutic process and potentially increasing adherence and motivation among patients undergoing amblyopia treatment. Roguelike games possess inherent factors such as random environment generation, nonmodality, complexity, and resource management. Additionally, roguelike mechanics have a high degree of compatibility with action shooting games. By leveraging these factors, different needs of various patients could be met and the game would achieve a high replayability, further improving the adherence. These characteristics align well with the cognitive abilities of patients with amblyopia and the repetitive training requirements of amblyopia treatment. This system not only adheres to the therapeutic principles of binocular treatment but also enhances patient engagement through the integration of gamified mechanisms.

### Adverse Events

In the safety profile of the Vision Planet binocular treatment, one participant developed progressive esotropia. The study included 2 participants with combined mechanism amblyopia. One child, diagnosed with intermittent exotropia, displayed a stable deviation posttreatment. The other child with esotropia experienced worsening deviations of 10 PDs in both near and far assessments, possibly due to overaccommodation induced by intensive and close visual training. The child had a consistent deviation of 10 PDs at all distances pretreatment. This was based on their stable alignment profile during the study, which we documented as E(T) in the table to acknowledge the intermittent nature of the deviation. It was presumed that a microtropia with anomalous retinal correspondence might have decompensated to reveal their true angle posttreatment. The improvement in SA for this child was modest, with a change of approximately one octave. Based on established literature, a change of around 2 octaves in the SA threshold is generally required to surpass the test-retest variability for most SA assessments [36]. Given that the change observed in this case was less than 2 octaves, we acknowledge that this improvement could fall within the bounds of normal test-retest variability. As such, while the child showed some improvements, we cannot definitively state that it reflects a true clinical gain beyond this variability. Further research is needed to explore the impact of binocular treatment on eye alignment.

### Limitations

This study has several limitations, including its small sample size, modest follow-up duration, lack of randomization, and absence of a control group. To more definitively evaluate the effectiveness of binocular treatment compared to other amblyopia treatments, a larger, multicenter randomized clinical trial using Vision Planet treatment is necessary. Another limitation is the use of the Titmus test for assessing SA, which includes monocular cues. It is worth noting, however, that participants who experience VA improvement are likely to become better at recognizing and interpreting these monocular cues compared to their initial performance. Despite these limitations, this study serves as an initial assessment of a new approach to treating amblyopia. Future research will need to include more comprehensive and structured clinical evaluations to further explore this treatment’s effectiveness. Additional studies and clinical trials are essential to confirm the efficacy and long-term benefits of this innovative treatment method.

### Conclusion

The Vision Planet treatment has shown positive effects on both DVA and SA in children aged 4-6 years with amblyopia, indicating the potential effectiveness of binocular digital therapy as a treatment option for unilateral amblyopia.

## Supplementary material

10.2196/63384 Multimedia Appendix 1 Vision Planet Questionnaire.

10.2196/63384 Multimedia Appendix 2 Compliance in 8 weeks.

## Abbreviations

BCVA: best corrected visual acuity
DVA: distance visual acuity
log: logarithm
logMAR: base 10 logarithm of the minimum angle of resolution
NVA: near visual acuity
PD: prism diopter
SA: stereoacuity
VA: visual acuity
