# Supplementary material for: Transformative Gamified Binocular Therapy for Unilateral Amblyopia in Young Children: Pilot Prospective Efficacy and Safety Study
Source: JMIR Serious Games. 2025 Jan 16;13:e63384. doi: 10.2196/63384 (PMC11756835; doi:10.2196/63384)
Supplement: Multimedia Appendix 1 [file games-v13-e63384-s001.docx]

| **Multimedia Appendix 1 Vision Planet Questionnaire** | | | | | |
| --- | --- | --- | --- | --- | --- |
| **Q1：Recommendation*** | | | **Q2：Treatment Preference** | | |
| How likely would you recommend *Vision Planet* to other amblyopic patients? | | | How likely would you choose *Vision Planet* over patching to treat amblyopia? | | |
| 10 | 6 | (54.5%) | Very likely | 1 | (9.1%) |
| 9 | 1 | (9.1%) | Likely | 6 | (54.5%) |
| 8 | 0 | (0.0%) | Neutral | 4 | (36.4%) |
| 7 | 1 | (9.1%) | Unlikely | 0 | (0.0%) |
| 6 | 3 | (27.3%) | Very unlikely | 0 | (0.0%) |
| 1-5 | 0 | (0.0%) |  |  |  |
| **Q3：User Friendliness** | | | **Q4：Satisfaction** | | |
| How user-friendly was the *Vision Planet*? | | | As a parent, what is your satisfaction level in the *Vision Planet*? | | |
| Very easy | 6 | (54.5%) | Very satisfied | 5 | (45.5%) |
| Easy | 4 | (36.4%) | Satisfied | 6 | (54.5%) |
| Neutral | 1 | (9.1%) | Neutral | 0 | (0.0%) |
| Difficult | 0 | (0.0%) | Dissatisfied | 0 | (0.0%) |
| Very difficult | 0 | (0.0%) | Very Dissatisfied | 0 | (0.0%) |
| **Q5：Integration in Life** | | | **Q6：Change in Appeal** | | |
| How did Vision Planet fit into your daily routine? | | | How was the appeal of the *Vision Planet* changed for your child? | | |
| Very easily | 2 | (18.2%) | Increasing | 2 | (18.2%) |
| Easily | 5 | (45.5%) | Decreasing | 0 | (0.0%) |
| Neutral | 3 | (27.3%) | Steady | 6 | (54.5%) |
| Difficult | 1 | (9.1%) | First increasing then decreasing | 3 | (27.3%) |
| Very difficult | 0 | (0.0%) | Stable | 0 | (0.0%) |
| **Q7：Monitor System** | | | *Recommendation scale was rated from 0 (not likely at all) to 10 (extremely likely) | | |
| How valuable did you find the ability to review child’s usage data on the system? | | |  |  |  |
| Very valuable | 5 | (45.5%) |  |  |  |
| Valuable | 6 | (54.5%) |  |  |  |
| Neutral | 0 | (0.0%) |  |  |  |
| Valueless | 0 | (0.0%) |  |  |  |
| Very valueless | 0 | (0.0%) |  |  |  |
