# Supplementary material for: Transformative Gamified Binocular Therapy for Unilateral Amblyopia in Young Children: Pilot Prospective Efficacy and Safety Study
Source: JMIR Serious Games. 2025 Jan 16;13:e63384. doi: 10.2196/63384 (PMC11756835; doi:10.2196/63384)
Supplement: Multimedia Appendix 2 [file games-v13-e63384-s002.docx]

| **Multimedia Appendix 2 Compliance in 8 Weeks** | | | |
| --- | --- | --- | --- |
| Start (0w) | End (8w) | Training time (h) | Completion rate (%) |
| 2023/6/28 | 2023/8/22 | 53.4 | 133.5% (53.4/40) |
| 2023/6/28 | 2023/8/22 | 45.7 | 114.2% (45.7/40) |
| 2023/7/21 | 2023/9/14 | 39.4 | 98.5% (39.4/40) |
| 2023/8/4 | 2023/9/28 | 36.4 | 91.0% (36.4/40) |
| 2023/8/11 | 2023/10/5 | 56.0 | 140.0% (56/40) |
| 2023/8/13 | 2023/10/7 | 45.9 | 114.8% (45.9/40) |
| 2023/8/13 | 2023/10/7 | 39.8 | 99.4% (39.8/40) |
| 2023/8/18 | 2023/10/12 | 17.1 | 42.7% (17.1/40) |
| 2023/9/1 | 2023/10/26 | 32.9 | 82.2% (32.9/40) |
| 2023/10/13 | 2023/12/7 | 38.9 | 97.2% (38.9/40) |
| 2023/10/13 | 2023/12/7 | 38.0 | 95.0% (38/40) |
